# Maternal Consumption of Non-Staple Food in the First Trimester and Risk of Neural Tube Defects in Offspring

**DOI:** 10.3390/nu7053067

**Published:** 2015-04-24

**Authors:** Meng Wang, Zhi-Ping Wang, Li-Jie Gao, Hui Yang, Zhong-Tang Zhao

**Affiliations:** 1 Department of Epidemiology and Health Statistics, School of Public Health, Shandong University, 44 WenhuaXilu Road, Jinan 250012, Shandong, China; E-Mails: mwang@cdc.zj.cn (M.W.); mycdc1234@163.com (Z.-P.W.); lijiegao@sdu.edu.cn (L.-J.G.); hyangsd1234@163.com (H.Y.); 2 Zhejiang Provincial Center for Disease Control and Prevention, 3399 Binsheng Road, Hangzhou 310051, Zhejiang, China

**Keywords:** case-control study, nutrition, neural tube defects

## Abstract

To study the associations between maternal consumption of non-staple food in the first trimester and risk of neural tube defects (NTDs) in offspring. Data collected from a hospital-based case-control study conducted between 2006 and 2008 in Shandong/Shanxi provinces including 459 mothers with NTDs-affected births and 459 mothers without NTDs-affected births. Logistic regression models were used to examine the associations between maternal consumption of non-staple food in the first trimester and risk of NTDs in offspring. The effects were evaluated by odds ratio (OR) and 95% confidence intervals (95% CIs) with SAS9.1.3.software. Maternal consumption of milk, fresh fruits and nuts in the first trimester were protective factors for total NTDs. Compared with consumption frequency of ˂1 meal/week, the ORs for milk consumption frequency of 1–2, 3–6, ≥7 meals/week were 0.50 (95% CI: 0.28–0.88), 0.56 (0.32–0.99), and 0.59 (0.38–0.90), respectively; the ORs for fresh fruits consumption frequency of 1–2, 3–6, ≥7 meals/week were 0.29 (95% CI: 0.12–0.72), 0.22 (0.09–0.53), and 0.32 (0.14–0.71), respectively; the ORs for nuts consumption frequency of 1–2, 3–6, ≥7 meals/week were 0.60 (95% CI: 0.38–0.94), 0.49 (0.31–0.79), and 0.63 (0.36–1.08), respectively. Different effects of above factors on NTDs were found for subtypes of anencephaly and spina bifida. Maternal non-staple food consumption of milk, fresh fruits and nuts in the first trimester was associated with reducing NTDs risk in offspring.

## 1. Introduction

Neural tube defects (NTDs) are a group of severe human congenital malformations caused by the incomplete closure of neural tube within about 28 days following conception [1]. NTDs are complex disorders and appear to be affected by multiple factors, with both genetic and environmental contributions. Folic acid and vitamin B12, as crucial factors for metabolic pathways, have been extensively studied and demonstrated the important roles in development of NTDs [2,3]. Since the introduction of folic acid fortification in staple food, many countries have reported declines in NTDs incidence overall [4,5,6,7,8,9]. However, a large number of NTDs cases still occur and the cause remains unclear. Thus, there is a need to research additional dietary factors, such as maternal non-staple food consumption in first trimester. In recent decades, studies have reported that intake of fresh fruits or vegetables, unpasteurized milk, pickled vegetables and caffeine during pregnancy may be associated with NTDs risk in offspring [10,11,12,13]. However, due to the different populations and food intake frequency in each study, the findings are not completely consistent without providing a clear conclusion. A previous study conducted by Piirainen *et* *al.* indicates that women may be more open to dietary change during pregnancy than at other times [14]. Considering the first trimester includes the critical window of neural tube closure, evaluating the effects of non-staple food consumption and proposing beneficial adjustment in maternal diets in the first trimester may have great public health implications.

Beginning in 2008, we performed a hospital-based case-control study in Shanxi and Shandong provinces to explore the risk factors of NTDs. In this study, we solicited the maternal non-staple food consumption information in the first trimester to investigate the potential associations with NTDs in offspring.

## 2. Methods

### 2.1. Participants

A hospital-based case-control study was conducted in Shandong and Shanxi provinces in China. According to differences of NTDs incidence, 18 counties in Shandong Province and 6 counties in Shanxi Province were randomly selected to serve as research sites. As eligible cases, we selected 459 mothers who gave birth to NTDs infants or whose NTDs-affected pregnancies were terminated based on the ultrasonic diagnosis within two years from January 2006 to December 2007. Then, from March to December 2008, we interviewed the participants to collect the relevant information face-to-face with the structured questionnaire. Based on ICD-10, in this study, NTDs included anencephaly (Q00.0), spina bifida (Q05) and encephalocele (Q01) complying with related defining features and diagnostic criteria specified in “Birth Defects Monitoring Manual of China”. The diagnosis was confirmed and the case was classified by full-time doctors. A total of 459 control mothers who gave birth to healthy infants in the same area, same hospital and within a week in the same year were enrolled. In particular, our study abided by the “Declaration of Helsinki” and the informed consents were obtained from the case and control mothers. This study was reviewed by research institutional review board of Shandong University and approved by the ethics committee (2006BA105A01).

### 2.2. Questionnaire

With the designed questionnaire, the interviewer solicited all the relevant information during the six months before until 3 months after conception of the participants face-to-face. The questionnaire design went through literature review, panel discussion, check and approval by experts, and revisions after pilot study. The same structured questionnaire was administered to each pair of mothers by the same one trained interviewer. Questionnaires were used to collect the information about mothers’ demographic characteristics, reproductive history and illness history, lifestyle behaviors, environmental hazardous substances exposure, nutritional status, diet adjustment, specific food consumption and the consumption frequency in the first trimester, use of folic acid from six months before until 3 months after conception. The contents of questionnaire have been described fully elsewhere [10]. Notably, the food frequency questionnaire has been tested and proved to be validated and tailored for a Chinese diet in the pilot study.

### 2.3. The Definition of Study Variables

During the interview, mothers were asked to report what they ate in the first trimester. In order to standardize the answers, the mothers were asked to read an enclosed list of food as a memory aid before replying. In this study, the non-staple food included meat, animal giblets, eggs, milk, legume, fresh vegetables, fresh fruits, nuts, pickled vegetable, alcohol and coffee. To access the above food consumption frequency in the first trimester, women were asked to estimate the food consumption frequency (≤1 meal per month, 2–3 meals per month, 1–2 meals per week, 3–6 meals per week, ≥7 meals per week).

In order to adjust the potential confounding factors, the following covariates were taken into consideration: maternal periconceptional occupation (farmer, factory worker, and business/officer/solider/technologist), annual per capita income (≤3600 ¥, 3600–7200 ¥, >7200 ¥), maternal education level (<middle school graduate, middle school graduate, >middle school graduate), maternal prepregnancy BMI (underweight, normal weight, overweight, and obesity), history of abortion or induced labor (yes, no), history of chronic disease before conception (yes, no), family history of birth defects (yes, no), maternal diet adjustment (yes, no), folic acid intake (never intake, periconceptional intake, within three months before conception intake, and within three months after conception intake.)

### 2.4. Statistical Analysis

Descriptive statistics were used to describe the demographic characteristics of subjects with frequency and proportion. Univariate conditional logistic regression analyses were carried out to evaluate effects of the covariates on the NTDs risk.

Univariate conditional logistic regression analyses were carried out to preliminarily determine which of the non-staple food consumption were associated with the risk of NTDs. In this part, the food consumption frequency was divided into two grades: ˂1 meal per week and ≥1 meal per week.

A multivariate conditional logistic regression model was constructed by adding all the significant factors from the univariate conditional logistic regression analyses and finally determined the food significantly associated with the risk of NTDs.

In order to further analyze each grade effect of the food on NTDs risk, a multivariate conditional logistic regression model was conducted. In this part, the food consumption frequency was divided into four grades: ˂1 meal per week, 1–2 meals per week, 3–6 meals per week and ≥7 meals per week. In addition, we constructed another three multivariate conditional logistic regression analyses to determine whether there were differences in the three major NTDs subtypes (anencephaly, spina bifida and encephalocele).

The effect values were reported by odds ratio (OR), with their 95% confidence intervals (95% CIs). The database was set up through EpiData3.1 and the data went through statistical process adopting analysis software SAS9.1.3.

## 3. Results

### 3.1. Social-Economic Characteristics of the Participants

A total of 459 pairs of participants were identified in the study. Among them, 259 pairs were from Shandong Province and 200 pairs were from Shanxi Province. Among total NTDs cases, there were 194 anencephaly cases (42.3%), 200 spina bifida cases (43.6%) and 65 encephalocele cases (14.1%), and there were no difference in NTDs types between the two provinces. Frequencies and proportions of social-economic characteristics among case and control mothers were shown in Table 1. Compared to control mothers, case mothers were more likely to be farmers, more likely to have lower annual per capita, education level, obese and more likely to have an abortion or induced labor, family history of birth defects, chronic disease before conception, not more likely to adjust diet in the first trimester and take folic acid supplementation periconceptionally.

Table 2 showed univariate analysis of the associations between weekly food consumption and NTDs risk. Compared to food consumption frequency ˂1 meal, the significant factors were meat consumption ≥1 meal, animal giblets consumption ≥1 meal, eggs consumption ≥1 meal, legume consumption ≥1 meal, milk consumption ≥1 meal, fresh vegetables consumption ≥1 meal, fresh fruits consumption ≥1 meal, nuts consumption ≥1 meal, picked vegetables consumption ≥1 meal.

Table 3 showed multivariate analysis of the associations between weekly food consumption and NTDs risk. Finally, food consumption of milk, fresh fruits and nuts entered the model. Compared to the food consumption frequency ˂1 meal per weekly, ORs for mothers who consumed milk, fresh fruits and nuts with the frequency ≥1 meal were 0.62 (95% CI = 0.42–0.93), 0.31 (95% CI = 0.14–0.70), and 0.62 (95% CI = 0.43–0.89).

**Table 1:** Demographic and obstetric characteristics of subjects and their relationship with neural tube defects (NTDs).

Factors	Cases (*N* *=* 459; *%*)	Controls (*N* *=* 459; %)	OR	95% CI
Maternal periconceptional occupation				
Farmer	349 (76.0)	329 (71.7)	*Ref.*	
Factory worker	57 (12.4)	35 (7.6)	1.46	0.91–2.33
Business/officer/soldier/technologist	53 (11.5)	95 (20.7)	0.46	0.30–0.70
Annual per capita income (¥)				
≤3600	232 (50.5)	175 (38.1)	*Ref.*	
3600–7200	159 (34.6)	164 (35.7)	0.64	0.46–0.89
>7200	68 (14.8)	120 (26.1)	0.35	0.24–0.53
Maternal education level				
<Middle school graduate	119 (25.9)	46 (10.0)	*Ref.*	
Middle school graduate	298 (64.9)	312 (68.0)	0.33	0.22–0.51
>Middle school graduate	42 (9.2)	101 (22.0)	0.14	0.08–0.24
Body mass index(BMI of mother)				
Normal	20 (4.4)	34 (7.4)	*Ref.*	
Underweight	313 (68.2)	334 (72.8)	1.64	0.92–2.95
Overweight	94 (20.5)	81 (17.6)	2.03	1.06–3.88
Obese	25 (5.4)	8 (1.7)	5.11	1.93–13.49
Family history of birth defects				
No	432 (94.1)	449 (98.0)	*Ref.*	
Yes	27 (5.9)	9 (2.0)	2.89	1.35–6.17
Abortion or induced labor				
No	244 (53.2)	280 (61.0)	*Ref.*	
Yes	215 (46.8)	179 (39.0)	1.38	1.06–1.80
Chronic disease before conception				
No	429 (93.5)	445 (96.9)	*Ref.*	
Yes	30 (6.5)	14 (3.1)	2.33	1.19–4.59
Diet adjustment in the first trimester				
No	251 (54.7)	131 (28.5)	*Ref.*	
Yes	208 (45.3)	328 (71.5)	0.27	0.20–0.38
Folic acid intake				
Never	326 (71.0)	243 (52.9)	*Ref.*	
Periconceptional folic acid intake ^a^	22 (4.8)	77 (16.8)	0.22	0.22–0.36
Within three months before conception folic acid intake ^b^	17 (3.7)	28 (6.1)	0.48	0.25–0.90
Within three months after conception folic acid intake ^c^	94 (20.5)	111 (24.2)	0.61	0.44–0.87

OR odds ratio, CI confidence interval, Ref reference; Percentages of each variable may not equal 100 because of missing data or rounding; ^a^: Take 0.4 mg of folic acid tablet daily and used across the time of 3 months before and 3 months after conception and continued for at least 1 month; ^b^: Take 0.4 mg of folic acid tablet daily and used 3 months before conception and continued for at least 1 month; ^c^: Take 0.4 mg of folic acid tablet daily and used 3 months after conception and continued for at least 1 month.

**Table 2:** Univariate analysis of the association between weekly food consumption and NTDs risk.

Weekly Food Consumption	Cases *N*	Controls *N*	OR	95% CI
Meat consumption				
˂1 meal	201 (43.8)	131 (28.5)	*Ref.*	
≥1 meal	257 (56.0)	328 (71.5)	0.43	0.31–0.60
Animal giblets consumption				
˂1 meal	378 (82.4)	321 (69.9)	*Ref.*	
≥1 meal	81 (17.6)	137 (29.8)	0.50	0.36–0.69
Eggs consumption				
˂1 meal	68 (14.8)	49 (10.7)	*Ref.*	
≥1 meal	391 (85.2)	410 (89.3)	0.65	0.42–0.99
Legume consumption				
˂1 meal	142 (30.9)	110 (24.0)	*Ref.*	
≥1 meal	115 (25.1)	92 (20.0)	0.70	0.52–0.94
Milk consumption				
˂1 meal	234 (51.0)	151 (32.9)	*Ref.*	
≥1 meal	224 (48.8)	308 (67.1)	0.41	0.30–0.55
Fresh vegetables consumption				
˂1 meal	15 (3.3)	2(0.4)	*Ref.*	
≥1 meal	444 (96.7)	457 (99.6)	0.13	0.03–0.58
Fresh fruits consumption				
˂1 meal	58 (12.6)	12 (2.6)	*Ref.*	
≥1 meal	401 (87.4)	446 (97.2)	0.16	0.08–0.33
Nuts consumption				
˂1 meal	266 (58.0)	184 (40.1)	*Ref.*	
≥1 meal	193 (43.0)	274 (59.7)	0.46	0.35–0.62
Picked vegetables consumption				
˂1 meal	267 (58.2)	323 (70.4)	*Ref.*	
≥1 meal	192 (41.8)	136 (29.6)	1.76	1.32–2.34

OR odds ratio; CI confidence interval; *Ref.* reference group.

**Table 3:** Multivariate analysis of association between weekly food consumption and NTDs risk.

Weekly Food Consumption	Case *N*	Control *N*	OR *	95% CI
Milk consumption				
˂1 meal	234 (51.0)	151 (32.9)	*Ref.*	
≥1 meal	224 (48.8)	308 (67.1)	0.62	0.42–0.93
Fresh fruits consumption				
˂1 meal	58 (12.6)	12 (2.6)	*Ref.*	
≥1 meal	401 (87.4)	446 (97.2)	0.31	0.14–0.70
Nuts consumption				
˂1 meal	266 (58.0)	184 (40.1)	*Ref.*	
≥1 meal	193 (42.0)	274 (59.7)	0.62	0.43–0.89

OR odds ratio; CI confidence interval; *Ref.* reference group; * ORs were adjusted for all variables listed in Table 1 and Table 2.

Table 4 further showed the specific effects of different food consumption frequency on the risk of NTDs, anencephaly and spina bifida respectively. For total NTDs, a 41%–50% reduction in risk of NTDs was observed at all levels of milk consumption (ORs ranged from 0.50 to 0.59 and the 95% CIs did not include the null). A 68%–78% reduction in risk of NTDs was observed at all levels of fresh fruits consumption (ORs ranged from 0.22 to 0.32 and the 95% CIs did not include the null). For anencephaly, a 94%–95% reduction in risk of NTDs was observed at all levels of fresh fruits consumption (ORs ranged from 0.05 to 0.06 and the 95% CIs did not include the null). For spina bifida, a 57%–83% reduction in risk of NTDs was observed at all levels of fresh fruits consumption (ORs ranged from 0.17 to 0.43 and the 95% CIs did not include the null).

**Table 4:** Effects of food consumption frequency on all NTDs, anencephaly, spina bifida and encephalocele.

Food Consumption Frequency	Case *N*	Control *N*	NTDs		Anencephaly		Spina Bifida	
			OR *	95% CI	OR *	95% CI	OR *	95% CI
Milk consumption								
˂1 meal per week	234 (51.0)	151 (32.9)	*Ref.*		*Ref.*		*Ref.*	
1–2 meals per week	48 (10.5)	57 (12.4)	0.50	0.28–0.88	1.64	0.63–4.26	0.17	0.07–0.43
3–6 meals per week	53 (11.5)	60 (13.1)	0.56	0.32–0.99	1.54	0.62–3.86	0.26	0.11–0.63
≥7 meals per week	123 (26.8)	191 (41.6)	0.59	0.38–0.90	1.20	0.58–2.48	0.43	0.21–0.88
Fresh fruits consumption								
˂1 meal per week	58 (12.6)	12 (2.6)	*Ref.*		*Ref.*		*Ref.*	
1–2 meals per week	54 (11.8)	44 (9.6)	0.29	0.12–0.72	0.06	0.01–0.49	0.46	0.11–1.86
3–6 meals per week	56 (12.2)	75 (16.4)	0.22	0.09–0.53	0.05	0.01–0.34	0.32	0.09–1.14
≥7 meals per week	291(63.4)	327 (71.2)	0.32	0.14–0.71	0.06	0.01–0.37	0.57	0.17–1.95
Nuts consumption								
˂1 meal per week	266 (58.0)	184 (40.1)	*Ref.*		*Ref.*		*Ref.*	
1–2 meals per week	73 (15.9)	95 (20.7)	0.60	0.38–0.94	0.94	0.44–2.00	0.31	0.14–0.69
3–6 meals per week	63 (13.7)	99 (21.6)	0.49	0.31–0.79	0.50	0.22–1.12	0.58	0.28–1.19
≥7 meals per week	57 (12.4)	80 (17.4)	0.63	0.36–1.08	0.29	0.11–0.81	0.94	0.42–2.10

OR odds ratio; CI confidence interval; *Ref.* reference group; * ORs were adjusted for all variables listed in Table 1 and consumption of milk, fresh fruits and nuts (≥1 meal and ˂1 meal).

## 4. Discussion

In this study, we found that maternal non-staple food consumption of milk, fresh fruits and nuts in the first trimester were associated with NTDs risk in offspring. Among them, maternal consumption of milk in the first trimester was associated with reducing the risk for total NTDs and spina bifida, but not for anencephaly. Fresh fruits consumption in the first trimester was associated with decreasing the risk for total NTDs and anencephaly, but not for spina bifida. With specific consumption frequency, nuts were associated with reducing risk of total NTDs (1–6 meals per week), subtypes of anencephaly (≥7 meals per week) and spina bifida (1–2 meals per week). In addition, our study also indicated that the preventive effect of milk weakened with the increased consumption frequency, both on total NTDs and spina bifida; moderate amount of fresh fruits consumption with frequency of 3–6 meals per week showed the strongest effects on total NTDs and anencephaly.

Milk contained many potentially NTDs-related nutrients, and vitamin B12 was one of them. Recently, studies have showed that low maternal vitamin B12 was a risk factor for NTDs [3,15,16]. Tucker *et al*. [17] first observed the vitamin B12 from milk was better absorbed than from meat. Naik *et al*. [18] further demonstrated that regular intake of milk could improve the vitamin B12 status among vegetarians with vitamin B12 deficient. In addition, studies suggested that dietary choline and betaine might help to prevent NTDs [19,20], and milk was one of the major food contributors [20,21]. However, due to the production methods, modern cow milk might have a high content of estrogen [22], which was reported associated with reducing serum folate availability [23]. In our study, the negative effects might partly explain why the preventive effect of milk on NTDs weakened with the increased consumption frequency in our study.

Preventive effects of fresh fruits for NTDs have been identified in many studies [24,25]. Consistent with previous studies, our results showed that fresh fruits consumption in the first trimester was associated with reducing risk of total NTDs and anencephaly. Regarding the effect of fresh fruits on spina bifida, our results indicated that fresh fruits were not associated with the risk of spina bifida. A previous study conducted by Yin *et al.* [26] confirmed our findings and reported no association between fresh fruits and spina bifida, although the population and consumption frequency were varied. However, a case-control study in Italy found that occasional consumption (less than 3 times a week) of fruits and vegetables was a risk factor for spina bifida [27]. In addition, our study presented that too much amount of fresh fruits consumption (≥7 meals per week) would weaken the NTDs preventive effect, which was never mentioned in other studies. An assumption we proposed was that too much fruits consumption might reduce other food intakes, such as meat, eggs or milk, by which affected the maternal diet quality during pregnancy eventually.

There were few studies on the association between nuts consumption in the first trimester and NTDs risk in offspring. In this study, we found that mothers consuming nuts with specific weekly frequency had a lower risk for total NTDs, subtypes of anencephaly and spina bifida. Although the biological mechanisms underlying the NTDs preventive effects of nuts were complicated and still remained unclear, our findings might be partly explained by the components of nuts. First, folate, a most important vitamin in preventing NTDs, was found to be abundant in nuts [28]. Second, nuts were good source of dietary myo-inositol, which had been observed associated with NTDs risk in offspring [29,30]. A study even proposed that myo-inositol soft gelatin capsules should be considered for the preventive treatment of NTDs in folate-resistant subjects [31].

Our study had several strengths. Good validity of cases classified by full time doctors with specified diagnostic criteria was helpful to minimize the misclassification of the participants. Besides, we evaluated the related factors for the three major NTDs subtypes, which would not obscure the potential associations that may be interfered by etiological heterogeneity.

Our study also had some limits. Firstly, with a retrospective design, our collected information from mothers up to 2 years after pregnancy, and recall bias could be a concern in our study. Secondly, in our study, the relatively small sample sizes limited our ability to study the association between non-staple food consumption in the first trimester and risk of the NTDs subtype of encephalocele. Thirdly, we did not collect the further information of non-staple food in study, such as milk type (unpasteurized milk or packet milk), the specific kind of fruits *et al*, which may affect the final results more or less. Fourthly, the short period of neural tube closure and diet adjustment after a mother realized she was pregnant might lead to exposure misclassification in our study. Finally, as there was bias resulted from some non-response mothers, the representative of the controls also could be a concern in this study.

In conclusion, the findings of the present study demonstrated that non-staple food of milk, fresh fruits, and nuts were associated with decreasing NTDs risk in offspring. Although the associations needed to be confirmed by further studies, we suggested that adequate intake of milk, fresh fruits and nuts were necessary for pregnant women in the first trimester.
